# Free radical homopolymerization of a vinylferrocene/cyclodextrin complex in water

**DOI:** 10.3762/bjoc.6.60

**Published:** 2010-06-01

**Authors:** Helmut Ritter, Beate E Mondrzik, Matthias Rehahn, Markus Gallei

**Affiliations:** 1 Institute of Organic Chemistry and Macromolecular Chemistry II, Heinrich-Heine-University of Duesseldorf, Universitaetsstrasse 1, D-40225 Duesseldorf, Germany; 2 Ernst-Berl-Institute for Technical and Macromolecular Chemistry, Technical University of Darmstadt, Petersenstrasse 22, D-64287 Darmstadt, Germany

**Keywords:** cyclodextrin, free radical polymerization, polyvinylferrocene, vinylferrocene

## Abstract

We report the radical initiated homopolymerization of a soluble vinylferrocene cyclodextrin-complex in water. Uncomplexed vinylferrocene **1** and the corresponding homopolymer are hydrophobic and completely insoluble in water. Complexation of **1** with methyl-β-cyclodextrin **2** results in clearly water-soluble structures due to incorporation of the ferrocene moiety into the cyclodextrin cavity. After free radical polymerization of the water-soluble complexed monomer, corresponding to polyvinylferrocene (PVFc), the water-soluble polymer is obtained due to the host guest interactions. Those polymeric complexes are stable in water up to about 90 °C. Above this temperature the polymer precipitates due to decomplexation. The complex was investigated by ^1^H NMR spectrometry, dynamic light scattering (DLS), differential scanning calorimetry (DSC), and lower critical solution temperature (LCST) measurements.

## Introduction

Since its discovery in 1951, ferrocene [1] and its derivatives and their applications have been the subject of numerous papers [2–7]. One derivative, vinylferrocene **1**, is of particular interest because of its anionic and radical polymerization behavior [8–12]. Due to its electrical semiconductivity properties the polymer of **1** has several practical applications. It is mainly used in biochemical sensors [13–14], batteries [15] and in the fuel industry [16]. However, the polymerization of the hydrophobic vinylferrocene in water has not been investigated.

Recently, we studied the radical polymerization of various vinyl monomers in water using cyclodextrin as the hydrophilic host component [17–25]. The hydrophobic part of the monomer molecule is included in the more hydrophobic cyclodextrin cavity yielding completely water-soluble complexes capable of polymerization. In this publication we wish to present our results of the aqueous free radical homopolymerization of vinylferrocene **1** as a guest in the host methyl-β-cyclodextrin (methyl-β-CD) **2**. The stability of the resulting complexed polymers in water is also reported.

## Results and Discussion

**Scheme 1:**
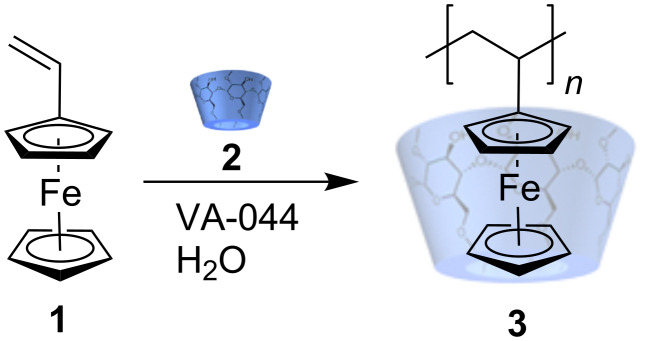
Homopolymerization of vinylferrocene **1**.

Vinylferrocene **1** was added to an aqueous solution of methyl-β-CD **2** and the complex obtained was subjected to radical polymerization in water at 50 °C. During the reaction an increase in viscosity was observed, due to the formation of complexed polyvinylferrocene **3**, which did not precipitate during the chain growth as a consequence of a strong host-guest interaction (Scheme 1). The complex remains in solution.

The ratio of insertion of vinylferrocene **1** and methyl-β-CD **2** in the complexed polymer **3** was analyzed by ^1^H NMR spectrometry by comparing the integrated signals of vinylferrocene **1** (singlets at 1.83 and 4.11 ppm, triplets at 4.25 and 4.44 ppm, doublet of doublets at 6.47 ppm) with the integrated signals of methyl-β-CD **2** (3.10–3.31, 3.45–3.65 and 4.75–5.30 ppm). From this data 1:1 complexation was indicated.

**Figure 1:**
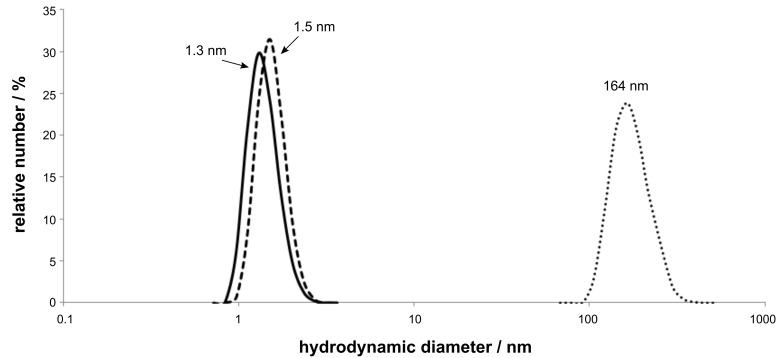
Dynamic light scattering of methyl-β-CD **2** (solid line), vinylferrocene **1** complexed with methyl-β-CD (dashed line) and complexed polyvinylferrocene **3** (dotted line).

The DLS results showed the hydrodynamic radii in water for molecular dispersed cyclodextrin **2** to be 1.3 nm. By comparison, the radius of the complex of monomer **1** with methyl-β-CD **2** is slightly increased at 1.5 nm. However, the complexed polymer **3** shows a significantly larger hydrodynamic radius of 164 nm (Figure 1).

The complexed polyvinylferrocene **3** was thermally stable up to about 90 °C in water, as demonstrated by turbidity measurements. The complex between methyl-β-CD **2** and polyvinylferrocene **4** remains stable almost up to boiling point of water. Heating above 90 °C, results in destabilization of the complex and causes the polymer to precipitate.

**Figure 2:**
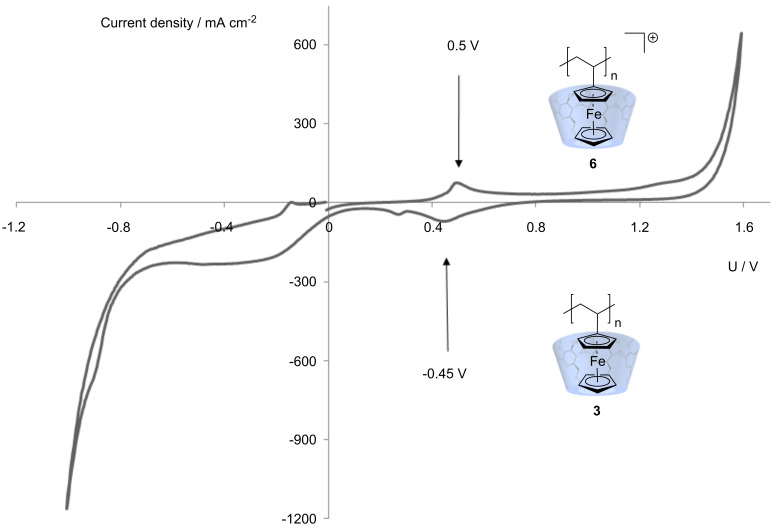
The cyclic voltammetry for the complexed PVFc/PVFc^+^-system **3**/**6**: 1.0 × 10^−3^ M substance in Na_2_SO_4_ | Ag/AgCl.

Furthermore, the reversibile redox behavior of complexed polyvinylferrocene **3** was evaluated by cyclic voltammetry. Oxidation of complexed polyvinylferrocene **3** to the polyvinylferricinium cation **6** takes place at 0.5 V and its reduction at −0.45 V (Figure 2).

**Figure 3:**
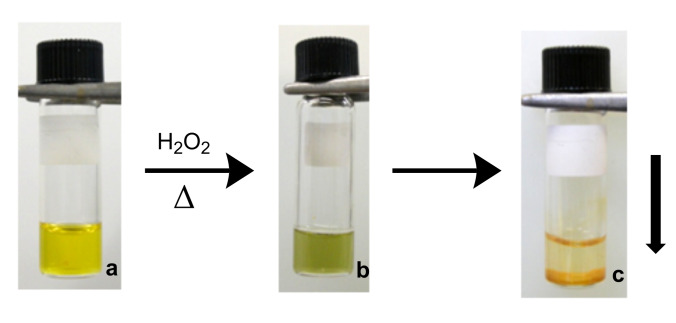
Redox behaviour of PVFc **3**: (a) the complexed PVFc **3** mixed with aqueous hydrogen peroxide solution was heated to 50 °C. The orange **3** oxidized to green PVFc^+^ **6** cation (b), which is reduced to the PVFc **4** and precipitated (c).

By contrast, irreversible oxidation occurred on heating the solution of complexed polyvinylferrocene **3** in aqueous hydrogen peroxide, which was accompanied with a change in color from yellow (Figure 3a) to green (Figure 3b). In a second step, the reduction of PVFc^+^ **6** to PVFc **4** was exothermic, hence the complexed polyvinylferrocene **3** was destabilized and the orange colored reduced form of polyvinylferrocene **4** precipitated, indicating that the individual ferrocene units along the chain no longer fit into the cavity of the cyclodextrin (Figure 3c).

In addition, when potassium 1-adamantane carboxylate was added as a guest molecule, which competes with the complexed homopolymer solution, the polymer precipitated due to the hydrophobic character of the uncomplexed ferrocene. This uncomplexed polyvinylferrocene was investigated by MALDI-TOF which indicated a mass of 6172.7 [M+Na]^+^.

**Scheme 2:**
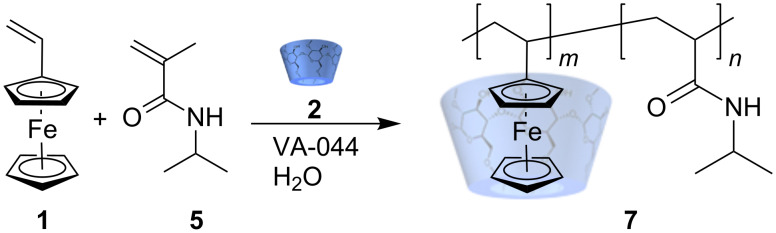
Copolymerization of vinylferrocene **1** with N*i*PAAM **5** (1:20).

Very recently a paper was published, reporting the expected LCST properties of the copolymer consisting of *N*-allylferrocenecarboxamide and *N*-isopropylacrylamide (poly(N*i*PAAM/FCN)) [26]. However, in this instance the copolymer was prepared by a classical synthesis in an organic solvent. Thus, we were encouraged to prepare a similar copolymer by free radical polymerization in water starting from cyclodextrin complexed vinylferrocene **1** and the water-soluble co-monomer *N*-isopropylacrylamide **5**, the latter in a 20 fold molar excess. The copolymer obtained (PVFc-co-P(N*i*PAAM)) **7** was water-soluble, since the cyclodextrin is not covalently attached to the ferrocene moiety (Scheme 2).

**Figure 4:**
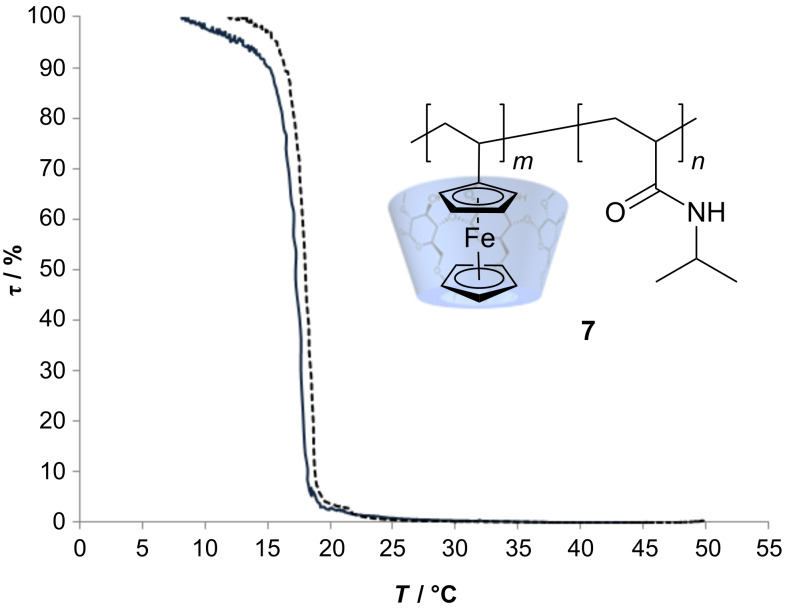
Lower critical solution temperature of PVFc-co-P(N*i*PAAM) **7** (1:20) (solid line): 17 °C and complexed N*i*PAAM with additional methyl-β-CD (dashed line): 19 °C.

The strong complexation was shown by correlations between methyl-β-CD **2** and polyvinylferrocene-co-poly(*N*-isopropylacrylamide) **7** at 4.16 ppm (cp) + 3.54 ppm (**2**) and at 4.17 (cp) + 3.52 ppm (**2**) in ^1^H^1^H ROESY-NMR spectrum. The LCST of the copolymer in water was slightly increased from 17 °C to 19 °C (Figure 4) after the addition of cyclodextrin.

## Conclusion

It can be concluded from the experiments described above that vinylferrocene can be easily polymerized in water after complexation with cyclodextrin. The strong polymer-CD complex obtained remained unchanged up to about 90 °C. The removal of cyclodextrin by the addition of a competing guest, potassium 1-adamantane carboxylate, was demonstrated. Additionally, the polymer shows redox behavior even in the presence of the cyclodextrin ring.

## Experimental Part

### Materials and Instrumentation

Methyl-β-cyclodextrin was obtained from Wacker-Chemie GmbH, Burghausen, Germany, and used after drying overnight with a vacuum oil pump over P_4_O_10_. Dimethyl-*d*_6_ sulfoxide (99.9 atom % D) was obtained from Euriso-Top SA, France. 2,2-Azobis[2-(2-imidazolin-2-yl)-propane] dihydrochloride (VA-044) was obtained from Cayman Europe, Estonia. *N*-Isopropylacrylamide (N*i*PAAM) was obtained from Aldrich, Germany. Vinylferrocene (VFc) was obtained from Sigma-Aldrich, Germany.

IR spectra were recorded with a Nicolet 5 SXB FTIR (Fourier transform infrared) spectrometer equipped with an ATR unit. The measurements were performed in the range of 4000–300 cm^−1^ at room temperature. ^1^H NMR spectra were recorded with a Bruker AC 500 at 20 °C. Chemical shifts were referenced to the solvent value δ 2.51 for dimethyl-*d*_6_ sulfoxide. Matrix-assisted laser desorption/ionization-time-of-flight mass spectrometry (MALDITOF-MS) was performed on a Bruker Ultraflex TOF mass spectrometer. Cyclic voltammetry measurements were performed on a simplot instrument with a resolution of <100 pA. Dynamic light scattering (DLS) experiments were carried out with a Malvern HPPS-ET at 25 °C. The particle size distribution was derived from a deconvolution of the measured intensity autocorrelation function of the sample by the general purpose mode algorithm included in the DTS software. DLS measurements were performed in water; the concentrations of the substances were 10 mg × mL^−1^. Each experiment was performed at least five times to obtain statistical information. Differential scanning calorimetry (DSC) measurements were performed on a Mettler DSC-30 instrument in a temperature range of −25 to 200 °C at a heating rate of 15 °C × min^−1^ as the average of five measurements using the midpoint method.

### Homopolymerization of vinylferrocene **1**

An aqueous solution of methyl-β-CD **2** in water (617.72 mg, 0.47 mmol) was prepared and purged with argon for 5 min. Vinylferrocene **1** (100 mg, 0.47 mmol) was added under an argon atmosphere and the mixture heated to 50 °C. VA-044 (1.53 mg, 4.7 × 10^−3^ mmol) was added to the mixture followed by the addition of a similar amount of the initiator after 8 h. After a reaction time of 48 h, the reaction mixture was freeze-dried. No residual monomer was evident by thin layer chromatography. According to differential scanning calorimetry (DSC), the obtained polyvinylferrocene **3** had a glass transition temperature (*T*_g_) of 145 °C.

FT-IR (film) **3**: 2902 cm^−1^ (–C–H), 1643 cm^−1^ (–C=C), 1452 cm^−1^ (CH_2_), 1153, 1033 cm^−1^ (–C–O), 1082 cm^−1^ (C–O–C), 964, 758, 702 cm^−1^ (=C–H); ^1^H NMR: (500 MHz, DMSO-*d*_6_, 298 K) **3**: δ (ppm) = 1.83 (CH_2_), 3.10–3.31 (14H, 5-H) 3.45–3.65 (28H, 6-H) 4.11 (s, 5H, 1-H), 4.25 (t, 2H, 2-H, ^3^*J* = 1.73 Hz), 4.44 (t, 2H, 3-H, ^3^*J* = 1.73 Hz), 4.75–5.30 (7H, 7-H) 6.47 (dd,1H, 4-H, ^3^*J* = 10.72 Hz (*cis*), ^3^*J* = 17.34 Hz (*trans*)).

### Copolymerization of vinylferrocene **1** and *N*-isopropylacrylamide **5**

An aqueous solution of methyl-β-CD **2** (617.72 mg, 0.47 mmol) was prepared and purged with argon for 5 min. Vinylferrocene **1** (100 mg, 0.47 mmol) was added under an argon atmosphere. After vinylferrocene **1** had completely dissolved, *N*-isopropylacrylamide (N*i*PAAM) **5** (1067.10 mg, 9.43 mmol) was added. The mixture was heated to 50 °C and VA-044 (32.02 mg, 0.10 mmol) added. After 3 h an orange solid precipitated.

FT-IR (film) **7**: 2970 cm^−1^ (–C–H), 1637 cm^−1^ (–C=C), 1533 cm^−1^ (–N–H), 1458 cm^−1^ (-CH_2_), 1385 cm^−1^ (–CH_3_), 1365 cm^−1^ (–C–N); ^1^H NMR: (500 MHz, DMSO-*d_6_*, 298 K) **7**: δ (ppm) = 1.05 (120H, 1-H), 1.46 (40H, 2-H), 1.98 (20 H, 3-H), 3.10–3.30 (42H, 11-H), 3.45–3.65 (84H, 12-H), 3.86 (20 H, 4-H), 4.25–3.96 (9 H, 7-H), 4.71–5.20 (21H, 10-H), 6.18 (1H, 6-H), 7.20 (20H, 5-H); MALDI-TOF **7**: *m*/*z* 1.6 × 10^4^.
